# Adsorption, thermodynamic, and quantum chemical investigations of an ionic liquid that inhibits corrosion of carbon steel in chloride solutions

**DOI:** 10.1038/s41598-022-16755-6

**Published:** 2022-07-22

**Authors:** Mohamed A. Abbas, Amr S. Ismail, K. Zakaria, A. M. El-Shamy, S. Zein El Abedin

**Affiliations:** 1 Petroleum Applications Department, Egyptian Petroleum Research Institute, P.B. 11727, Nasr City, Cairo Egypt; 2 Petrochemicals Department, Egyptian Petroleum Research Institute, P.B. 11727, Nasr City, Cairo Egypt; 3 Analysis and Evaluation Department, Egyptian Petroleum Research Institute, P.B. 11727, Nasr City, Cairo Egypt; 4 Electrochemistry and Corrosion Laboratory, Physical Chemistry Department, National Research Centre, Dokki, 12622 Cairo Egypt

**Keywords:** Materials science, Mathematics and computing

## Abstract

The purpose of this work lies in the use of ionic liquids as corrosion inhibitors due to the difficulty in some oil fields with the solubility of corrosion inhibitors and these materials can be miscible with water and thus provide a solution to such problems in the industry. The second purpose is concerned with the lower toxicity of these compounds compared with the most common corrosion inhibitors. The study covered the corrosion inhibition performance of the ionic liquid 1-butyl-3-methylimidazolium trifluoromethyl sulfonate ([BMIm]TfO) for carbon steel in 3.5% NaCl solutions. The study comprised electrochemical, adsorption, and quantum chemical investigations. The results manifested that [BMIm]TfO can be considered a promising corrosion inhibitor and the inhibition efficacy intensifies as the concentration rises. The observed inhibitive effect can be correlated to the adsorption of the ionic liquid species and the creation of protecting films on the surface. The mode of adsorption follows the Langmuir adsorption isotherm. The polarization results showed that the ionic liquid [BMIm]TfO functions as a mixed inhibitor. Reliance of the corrosion influence on the temperature in the existence and absence of [BMIm]TfO was demonstrated in the temperature range of 303–333 K using polarization data. Activation parameters were determined and discussed. The observed inhibition performance of [BMIm]TfO was correlated with the electronic properties of the ionic liquid using a quantum chemical study.

## Introduction

Corrosion is a serious and extremely costly problem especially in petrochemicals and petroleum production operations^1,2^. Besides the high potential to employ more advanced corrosion-resistant components, carbon steel tills are the main fabricated material used for the huge pipeline’s construction in the different crude oil processing stages such as extraction, transmission, and storage Carbon steel pipes have been widely employed in transporting gases and liquids^3,4^. One of the most aggressive surroundings in petroleum production and oil refining processes is the saline water environment which contains different amounts of dissolved salts, especially sodium chloride^5,6^. The protective oxide film created on the surface can deteriorate in the existence of chloride ions based on an aggressive environment^7,8^.

Different corrosion mitigation regimes can be applied to reduce the harmful corrosion impacts. Among the conventional corrosion mitigation techniques, the utilization of eco-friendly inhibitors has advantages such as, e.g., high feasibility, high efficiency, and minimization the environmental hazards^9^. In recent times, ionic liquids were proven to be effective, eco-friendly inhibitors for the corrosion of different types of metals and alloys in corrosive environments such as basic, acidic, or salty solutions^10,11^. Due to their bulky structure and the presence of heteroatoms, ionic liquids are regarded as potential corrosion inhibitors^12^.

Ionic liquids, in general, have a promising corrosion inhibition tendency due to their capacity for the adsorptive contact with the surface, which forms protective layers against corrosive media. In general, the majority of ionic liquids exhibit this tendency. The types of cations and anions present in ionic liquids have a direct bearing on the levels of adsorption and inhibition efficiency that may be achieved with those liquids. In a recent study, we demonstrated that the ionic liquid 1-butyl-1-methylpyrrolidinium trifluoromethyl sulfonate ([BMP]TfO) may serve as a bi-functional inhibitor for both the corrosion of carbon steel in chloride solutions and the growth of bacteria. [BMP]TfO inhibits both types of corrosion^13^. It was also shown that the ionic liquids 1-(2-hydroxyethyl)-3-methylimidazolinium chloride and 1-ethyl-3-methyleimidazolinium chloride are effective corrosion inhibitors and biocides. These findings were published in the journal Ionic Liquids^14^. It was shown that the integration of the hydroxy group in the imidazolium cation enhances the corrosion inhibition impact^15^. In this paper, the corrosion inhibition performance of the ionic liquid [BMIm]TfOfor carbon steel in 3.5% NaCl solutions was demonstrated. The study comprised electrochemical, adsorption, and quantum chemical investigations.

## Materials and methods

### Materials

High purity [BMIm]TfO, was obtained from Io.Li.Tec.Co., Germany, and it was used as received. Carbon steel specimens having dimensions of (5.6 cm L, 2.7 cm W, and 0.5 cm T) were used for the standard procedures of the corrosion measurements. Before use, the steel coupons were abraded with proper emery papers to give a homogeneous surface. An aggressive solution of 3.5% NaCl was prepared as a simulated corrosive environment. Different concentrations of the employed ionic liquid, 25, 50, 100, 200, and 500 ppm, were used, and various temperatures of 303, 313, 323, and 333 K, were applied.

### Methods

For the electrochemical measurements, a common cell, fitted with a reference electrode—saturated calomel reference electrode (SCE) a counter electrode (CE), platinum, and the working electrode (WE), carbon, steel, was utilized. All electrochemical tests were performed employing a Volta lab 40 Potentiostat PGZ 301. Polarization experiments were carried out by sweeping the electrode potential at a rate of 2 mVs^−1^ and recordings the current. All polarization data were fitted using the fitting tools implemented in Volta Master 4 software by Tafel extrapolation method. The frequency range of 100 kHz–0.05 Hz with an amplitude peak of 10 mV using an AC signal was applied for the electrochemical impedance measurements. The impedance curves were fitted using the ZSimpWin3.60 software. For weight loss measurements, rectangular-shaped steel specimens with dimensions of 3 cm × 5 cm × 0.05 cm were used. The immersion time was 6 h in 3.5% NaCl at different temperatures in the absence and presence of different concentrations of [BMIm]TfO.

A scanning electron microscope, JEOL model JSM-53000, was employed for investigating the morphology of the electrodes. The quantum chemical investigations were executed using MP2 functional with a 3–21 G (d,p) basis set. The energies of high occupied molecular orbital (E_HOMO_) and low unoccupied molecular orbital (E_LUMO_) were estimated. Furthermore, the energy gap between LUMO and HOMO (ΔE) was determined^16^.

## Results and discussions

### Potentiodynamic polarization

**Figure 1:**
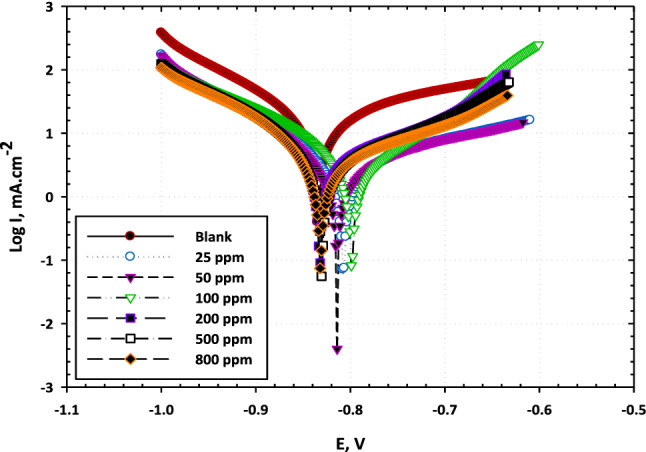
Potentiodynamic polarization curves for the carbon steel in a 3.5% NaCl medium with and without different concentrations of [BMIm]TfO.

The curves of Fig. 1 show potentiodynamic polarization responses of carbon steel in 3.5% NaCl solutions free and containing different amounts of [BMIm]TfO. The parameters obtained from the polarization curves, corrosion currents and potentials (i_corr_, E_corr_, respectively), corrosion rate (CR), inhibition efficiency based on corrosion current density (EF_icorr_%), and efficiency based on the corrosion rate (EF_CR_ %), cathodic Tafel slope (b_c_), and anodic Tafel slope (b_a_) are presented in Table 1. As shown in Fig. 1, the polarization curve recorded in absence of [BMIm]TfO exhibits an active trend as the current steeply rises with sweeping the potential. In addition to the ionic liquid inhibitor, a slight impact was noticed on the cathodic branch of the curve. However, a pronounced effect was recorded on the anodic branch. Both the cathodic and anodic curves shift to more negative and more positive potentials, respectively, on the addition of the [BMIm]TfO revealing the inhibiting influence.

**Table 1:** Polarization parameters obtained for carbon steel in 3.5% NaCl-free and containing [BMIm]TfO.

Concentration	Parameter							
	E_corr_ mV	i_corr_ mA/cm^2^	b_a_ mV/dec	b_c_ mV/dec	CR µm/Y	θ	EF_icorr_%	EF_CR_%
3.5% NaCl	− 834	0.016	259	− 131	185			
25 ppm	− 805	0.0069	262	− 147	81	0.568	56.8	56.3
50 ppm	− 813	0.0066	202	− 140	78	0.59	59	57.8
100 ppm	− 798	0.0063	185	− 148	74	0.606	60.6	60
200 ppm	− 833	0.0059	123	− 164	70	0.631	63.1	62.2
500 ppm	− 830	0.0044	384	− 128	52	0.726	72.6	71.8
800 ppm	− 832	0.0042	327	− 202	50	0.75	75	72.9

As the content of [BMIm]TfO increases, the current density decreases, and subsequently the corrosion rate reduces Table 1. The highest inhibition efficiency was 75% for the inhibitor concentration of 800 ppm. The observed inhibition influence is due to the creation of a protecting layer because of the adsorption of the ionic liquid species on the electrode surface. This layer isolates the surface from the corrosive environment which, in turn, leads to the observed inhibition. The extent of covering the surface (Ɵ) [BMIm]TfO was estimated. The degree of surface coverage rises as the content of [BMIm]TfO in the electrolyte increases. Full coverage of the electrode surface by the ionic liquid species was not reached in the studied concentration range of [BMIm]TfO.

**Figure 2:**
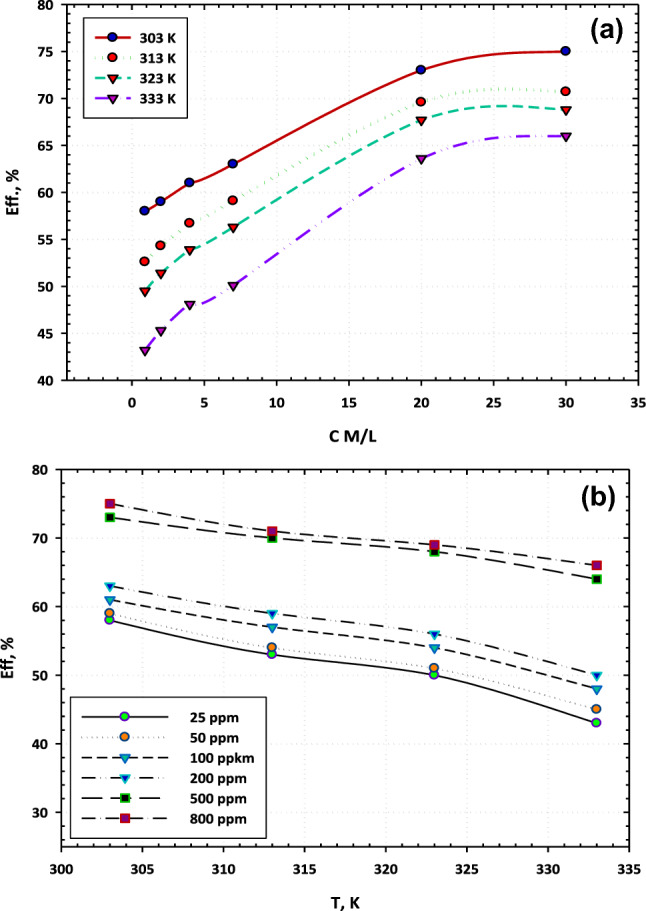
The dependence of the inhibition efficiency of [BMIm]TfO on the corrosion of carbon steel in 3.5% NaCl solutions as a function of (**a)** temperature and (**b**) concentration.

The dependence of the corrosion inhibiting efficacy of [BMIm]TfO on the concentration and temperature was explored. Figure 2a manifests the change in the inhibiting efficiency with the concentration of [BMIm]TfO at various temperatures. As seen, the inhibiting efficacy rises as the [BMIm]TfO concentration increases for all temperatures. Figure 2b displays the dependence of the inhibition efficiency on the temperature of the electrolyte at different concentrations of [BMIm]TfO. It is seen that at one, and the same concentration of [BMIm]TfO, the corrosion inhibition efficacy decreases as the temperature increases. At higher concentrations of [BMIm]TfO, the extent of the negative influence of the temperature on the inhibition efficiency shrinks^17^.

### Electrochemical impedance spectroscopy

**Figure 3:**
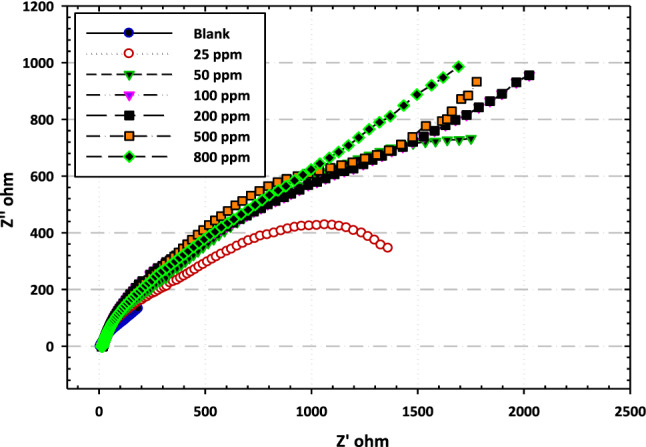
Typical Nyquist plots for the carbon steel in 3.5% NaCl without and with different concentrations of [BMIm]TfO.

The corrosion inhibition performance of [BMIm]TfO was investigated using the electrochemical impedance spectroscopy (EIS) technique. Figure 3 exhibits the Nyquist plots of the carbon steel electrode in the test electrolyte-free and containing different concentrations of [BMIm]TfO. As manifested in Fig. 3, the plots show imperfect capacitive loops. The higher the loop diameter always reveals stop corrosion resistance^18^. With the addition of [BMIm]TfO, the diameter increases signifying its inhibiting effect on the corrosion of carbon steel in the test electrolyte. This is attributable to the adsorption and formation of a protecting film of the ionic liquid on the electrode surface. This film masks the surface, leading to the lessening of the corrosion attack as evidenced by the reduction of the estimated double layer capacitance C_dl_, Table 2. Also, the values of (R_S_), (R_P1_), and (R_P2_) increase as the content of [BMIm]TfO increases^19^.

**Table 2:** EIS parameters for carbon steel in 3.5% NaCl free and containing [BMIm]TfO.

Conc	Parameters										
	Rs (ohm cm^2^)	Q (S s^n^ /cm^2^) × 10^–6^	n	R_pors_ (ohm.cm^2^)	C_coat_ (mF/cm^2^)	Q (S s^n^ /cm^2^)10^–6^	n	R_ct_ (ohm cm^2^)	C_dl_ (mF/cm^2^)	θ	EF%
3.5% NaCl	14.6	4307	0.65	246	4.44	2000	0.68	1165	2.97		
25 ppm	7.96	1785	0.74	357	1.52	718	0.66	1920	0.84	0.393	39.3
50 ppm	7.31	1270	0.79	490	1.12	701	0.76	2025	0.78	0.425	42.5
100 ppm	3.97	1100	0.82	593	1.001	576	0.8	3093	0.66	0.623	62.3
200 ppm	6.8	923	0.8	747	0.84	435	0.83	3692	0.47	0.685	68.5
500 ppm	1.6	756	0.82	792	0.67	266	0.8	3963	0.26	0.706	70.6
800 ppm	5.8	530	0.76	873	0.42	155	0.57	4628	0.12	0.749	74.9

**Figure 4:**
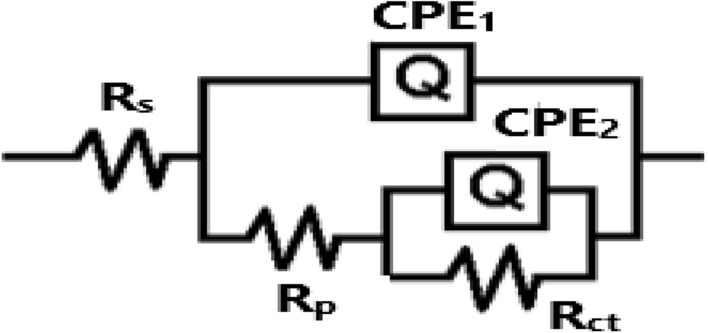
Equivalent circuit model fitted the electrochemical impedance spectroscopy (EIS) data.

The equivalent circuit used to fit the Nyquist plots is shown in Fig. 4. The values of the estimated EIS parameters are compiled in Table 2. The parameters include the solution resistance (R_s_), the constant phase element (Q), the polarization resistance between the surface and the formed film during the immersion in the test electrolyte (R_p1_), the capacitance of double layer (C_dl_), and the corrosion resistance at the surface film/electrolyte interface (R_p2_).

### Adsorption isotherms

Adsorption isotherm studies were performed to explore the nature of the interaction of the ionic liquid species with the carbon steel surface. Several adsorption isotherms can describe the relationship between the extent of surface coverage and the content of adsorbed species such as Langmuir, Freundlich, and Temkin isotherms. The adsorptive interaction of [BMIm]TfO was found to obey Langmuir isotherm. The Langmuir adsorption isotherm can be defined in the following Eq. (1)^20^.

1 $$\frac{{C}_{inb}}{\theta }=\frac{1}{{k}_{ads}}+{C}_{inb}$$

where (θ) is the surface coverage degree (obtained previously from EIS and PDP measurements), (C_inb_) is the concentration of [BMIm]TfO, and (K_ads_) is the adsorption equilibrium constant.

**Figure 5:**
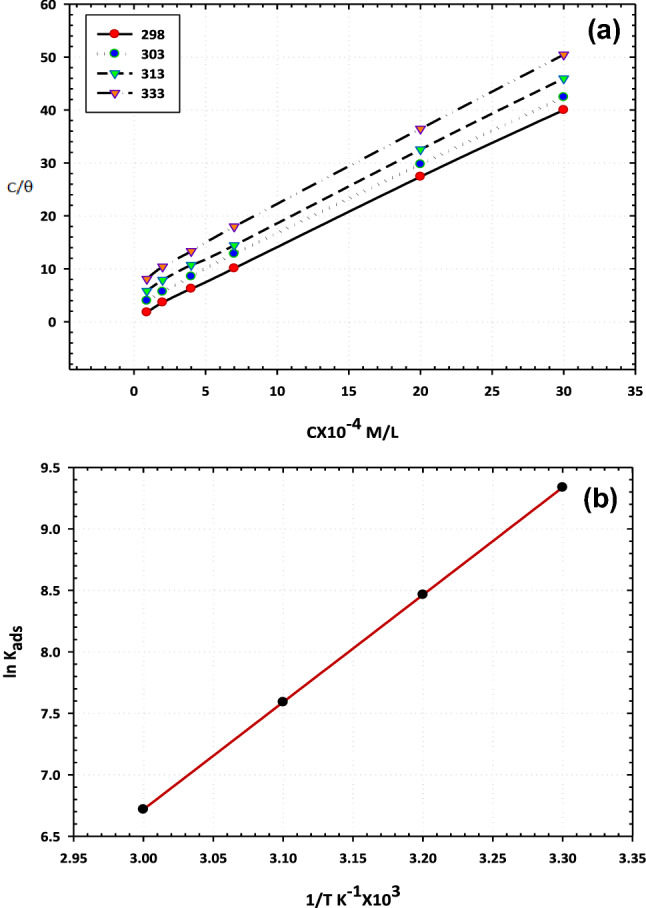
Langmuir (**a**) and Temkin (**b**) isotherms for adsorption of the ionic liquid on the carbon steel in 3.5% NaCl solutions.

Plotting the Langmuir relation between C_inb_/θ and C_inb_ gives significant straight lines with a correlation coefficient of unity and an average slope value of 1.35, Fig. 5a. Thus, it is evidence that the ionic liquid species are adsorbed in absence of any side interactions^18^. The equilibrium constant for [BMIm]TfO adsorption (K_ads_) was calculated using the intercept of the C_inb_/θ versus C_inb_ line, Table 3. Consequently, K_ads_ value was employed to estimate the adsorption standard free energy (ΔG°_ads_) based on the Eq. (2):

**Table 3:** Adsorption isotherms parameters.

T, K	R^2^	Slope	K_ads_, (M^–1^)	∆G^o^_ads_, (KJ mol^−1^)	∆H^o^_ads_, (KJ mol^−1^)	∆S^o^_ads_, (J mol^−1^ K^−1^)
303	0.9998	1.3	11,061	− 34	− 73	− 162
313	0.9996	1.3	3145	− 31		
323	0.9998	1.4	2014	− 31		
333	0.9995	1.4	1348	− 31		

2 $${K}_{ads}=\frac{1}{55.5}\mathrm{exp}(-\frac{{\Delta G}_{ads}^{o}}{RT})$$

where R, T, and 55.5 are gas constant, the temperature of the system, and water molecules concentration in molar. The values of ∆Goads were determined for the ionic liquid inhibitor at different temperatures as shown in Table 3. It is seen that the high negative values of ∆G^o^_ads_ are correlated to the impulsive adsorption performance of [BMIm]TfOon the surface and refer to the steadiness state of the adsorbed layer. This behaviour indicates the intensive interaction between the ionic liquid constituents and the electrode surface^21^. As a common concept, the values of ∆G^o^_ads_ up to − 20 kJ mol^−1^ are associated with the electrostatic interaction of the charged compounds with the charged surface in which the adsorption process, in this case, is only physical. With increasing the values of ∆G^o^_ads_ above − 40 kJ mol^−1^ the chemical adsorption will be found^22^.

From the obtained measurements, the values of ∆G^o^_ads_ were stabilized at − 31 kJ mol^−1^ in the temperature range from 303 to 333 K. This indicates that the adsorption of ionic liquid species is typical physical sorption. The results reveal that the adsorption of the species of the ionic liquid, typically the imidazolium cation, occurs physically, electrostatically, via the interaction between the imidazolium cation and the charged centers on the electrode surface. The spontaneous nature of the adsorption of the ionic liquid species on the surface is indicated by the negative values of ∆G^o^_ads_^23^. The heat of adsorption ∆H^o^_ads_ was also estimated from the Van’t Hoff Eq. (3):

3 $$ \ln \;{\text{K}}_{{{\text{ads}}}} = - \frac{{{\text{H}}_{{{\text{ads}}}}^{0} }}{{{\text{RT}}}} + {\text{A}} $$

By plotting the relation between ln K_ads_ against 1/T a straight line is obtained as manifested in Fig. 5b. The slope of the obtained line is equivalent to (− ∆H^o^_ads_/R) in which the adsorption heat value (∆H^o^_ads_) is nearly the standard adsorption heat value under the testing procedures^20^. From the thermodynamics, the standard entropy of adsorption (∆S^o^_ads_) can be obtained using the Eq. (4):

4 $$ \Delta {\text{S}}_{{{\text{ads}}}}^{0} = - \frac{{\Delta {\text{H}}_{{{\text{ads}}}}^{0} - \Delta {\text{G}}_{{{\text{ads}}}}^{0} }}{{\text{T}}} $$

The calculated thermodynamic parameters (∆G^o^_ads_, ∆H^o^_ads_ and ∆S^o^_ads_) are compiled in Table 3. It is seen that the ∆H^o^_ads_ value is negative (− 73 KJ mol^−1^) indicating the impact of [BMIm]TfO adsorption on the surface. By inspection of the values of (∆S^o^_ads_) as listed in Table 3, it is obvious that the ∆S^o^_ads_ value is the negative sign (− 162 J mol^−1^ K^−1^). The negative signal of ∆S^o^_ads_ can be correlated to dissolute medium which is generally proved as a growth in the disorder, as the reactants are turning into efficient complexes. Moreover, the observed manner can be explained by the substitution mechanism of more water molecules throughout the adsorption process of ionic liquid on the surface^24^.

### Kinetic activation

Additional to the thermodynamic investigations, the kinetic activation model is also an important technique to study the inhibition mechanism and explain the features of the protective action of [BMIm]TfO at different temperatures. Arrhenius equation was employed to estimate the activation parameters of the corrosion see Eq. (5)^25^:

5 $$ \ln {\text{CR}} = - \frac{{{\text{E}}_{{\text{a}}} }}{{{\text{RT}}}} \times \ln {\text{A}} $$

where, (CR) represents the corrosion rate, (E_a_) is the energy of activation, (R) is the gas constant, and (A) is the pre-exponential factor.

**Figure 6:**
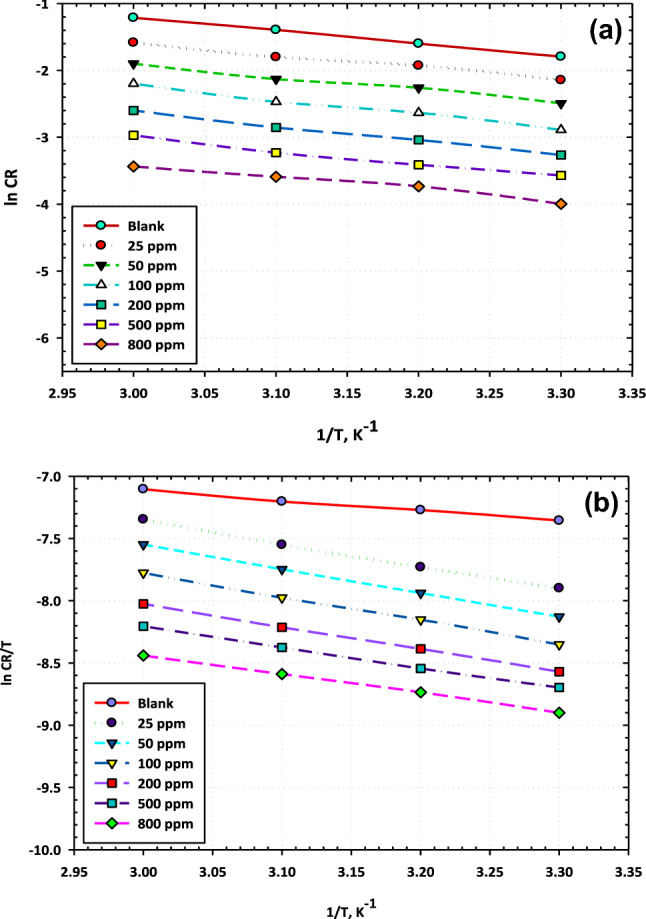
(**a**) Arrhenius and (**b**) Transition-state plots for carbon steel corrosion in 3.5% NaCl without and with different concentrations of [BMIm]TfO.

The Arrhenius plots of the relation between the ln (CR) and 1/T are depicted in Fig. 6a. As seen, a linear behavior is obtained and the slopes of the straight lines is (− E_a_/R) hence, the values of the activation energy (E_a_) in the presence and absence of various concentrations of [BMIm]TfO were determined. Moreover, there is another formulation for the transition state as described in the following Eq. (6)^26^.

6 $$ \ln {\text{CR}} = - \frac{{{\text{RT}}}}{{{\text{Nh}}}}\exp \left\{ {\frac{{\Delta {\text{S*}}}}{{\text{R}}}} \right\}\exp \left\{ {\frac{{ - \Delta {\text{H*}}}}{{{\text{RT}}}}} \right\} $$

The terms of the equation are, Plank’s constant (h), Avogadro’s number (N), the activation entropy (∆S^*^), and the activation enthalpy (∆H^*^). The plots of ln CR/T versus 1/T for the ionic liquid inhibitor are presented in Fig. 6b. The slope of the obtained lines equals (−∆ H^*^/R) and the intercept is (lnR/Nh + ∆S^*^/R), hence the values of ∆H^*^ and ∆S^*^can be estimated. At definite concentration ranges of the ionic liquid inhibitor, the activation parameters for carbon steel in sodium chloride medium are gathered and illustrated in Table 4. The results reveal that with the existence of [BMIm]TfO the activation energy rises, and the activation enthalpy slightly reduces meanwhile the values of the entropy for the corrosion process significantly rise. The energy of activation (Ea), which is the minimal amount of energy required to launch a chemical reaction, reaches a value of 24 kJ mol^−1^ at a high ionic liquid content and for the blank solution is only 16 kJ mol^−1^. The increase of the E_a_ value under the effect of ionic liquid inhibitor might be attributed to the physical sorption regime^27^. Additional explanation reported that the raising of the activation energy value might also be attributable to the gradual reduction in the adsorption process of [BMIm]TfO on the surface under the effect of heat. Based on these phenomena, as the adsorption process reduces more desorption action of ionic liquid species occurs as the protection and dissolution systems are in balance. It is also revealed that the sequential values of E_a_ and ∆H^o^ as presented in Table 4 are varied in the same trend confirming the common thermodynamic phenomena. On particular monitoring, it has been observed that the value of the activation entropies is negative signifying that the activated complex is in the rate-determining stage and demonstrating combination rather than separation^28^.

**Table 4:** Activation energy parameters at different concentrations of [BMIm]TfO.

Conc., ppm	E_a_, (KJ mol^−1^)	∆H^o^_a_, (KJ mol^−1^)	∆S^o^_a_, (J mol^−1^ K^−1^)
3.5% NaCl	16	7	− 236
25	15	15	− 213
50	16	16	− 212
100	19	16	− 215
200	20	15	− 219
500	21	14	− 225
800	24	13	− 230

### Surface analysis

**Figure 7:**
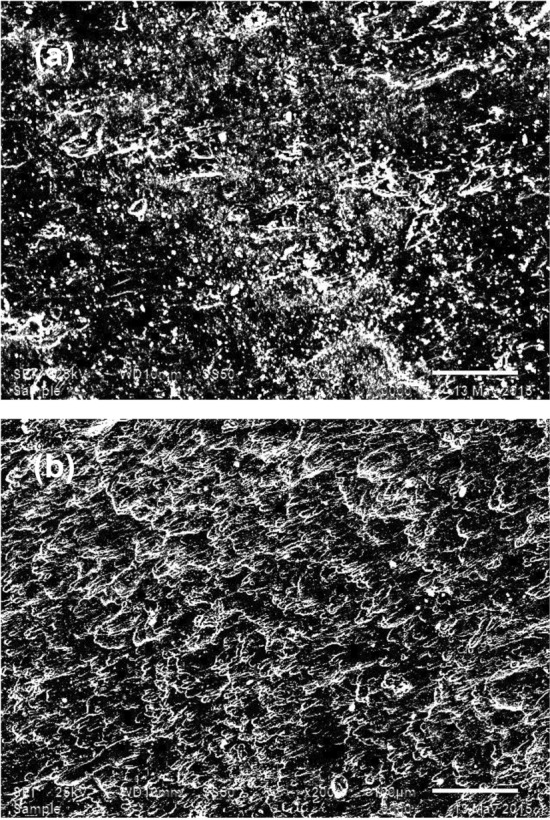
SEM images of (**a**) polished carbon steel, and (**b**) after 24 h of immersion in 3.5% NaCl and [BMIm]TfO.

The utilization of surface analytical techniques is quite significant for realizing the characteristics of materials, especially the metallic surfaces after exposure to the various aggressive environments. SEM–EDX examination of the electrode surface following dipping in the test electrolytes was performed. The SEM images of Fig. 7a and b show the surface after the exposure to3.5% NaCl without and with the addition of [BMIm]TfO, respectively. Figure 7a representative the SEM image of the uninhibited steel surface shows a deteriorated, rough surface as a result of the corrosion^29^.

In the absence of [BMIm]TfO the whole surface of the carbon steel is usually concealed with the corrosion products. The porous corrosion product layer is unable to protect the surface from extreme corrosion. The addition of the ionic liquid inhibitor to the test electrolyte reduced the aggressive attack on the steel surface as revealed in Fig. 7b. The surface is relatively free from the corrosion products and compared with the uninhibited sample the surface is smoother signifying the inhibiting action of [BMIm]TfO^30^.

**Figure 8:**
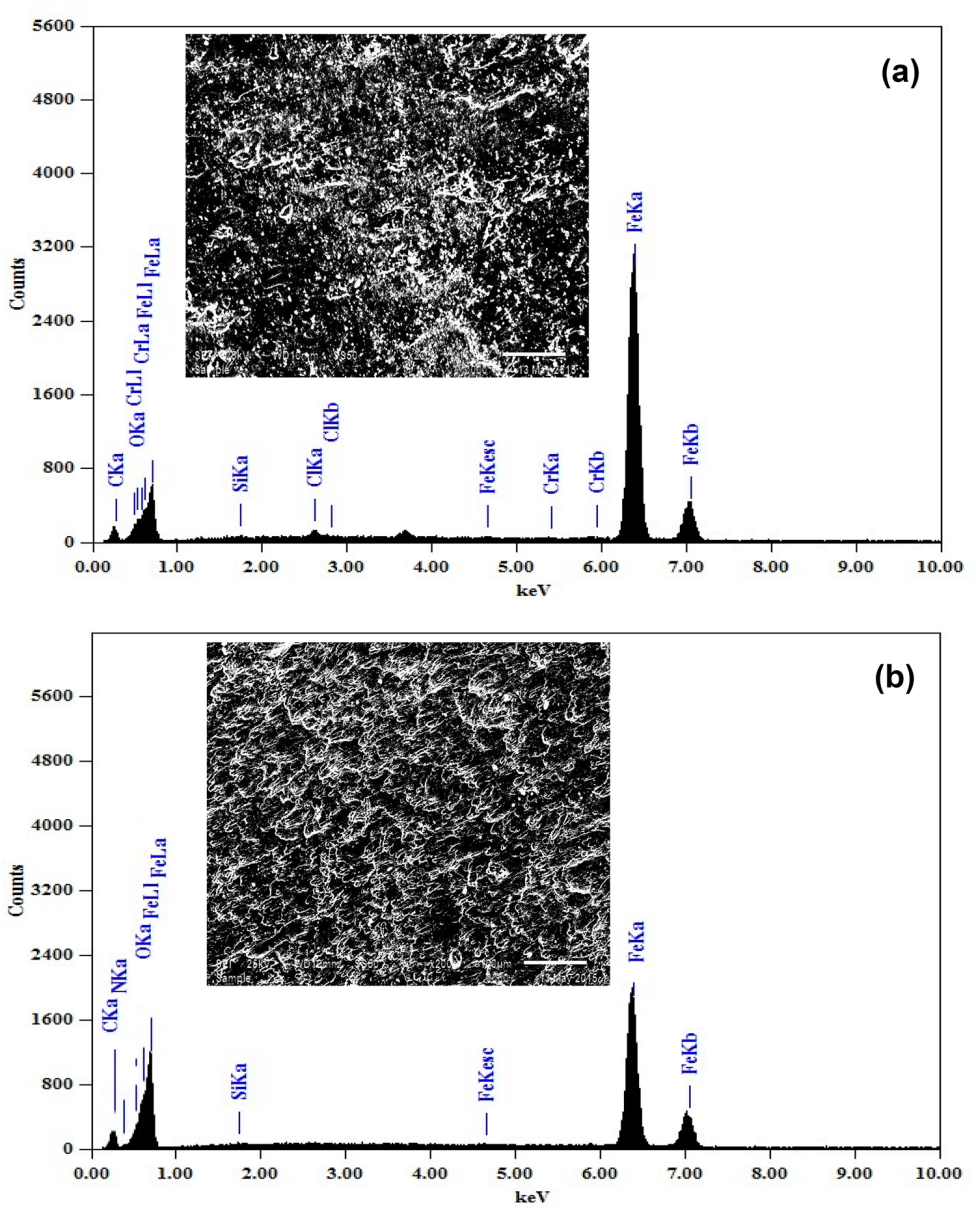
EDX spectra of (**a**) polished carbon steel, (**b**) after 24 h of immersion in 3.5% NaCl and [BMIm]TfO.

Figure 8 represents the EDX spectrum of the uninhibited sample Fig. 8a and inhibited sample Fig. 8b since it shows confirms the SEM results as, in addition to the Fe, O, and C peaks, a peak of N is recorded owing to the adsorption of the imidazolium cation leading to the observed corrosion inhibition. Furthermore, under the continuation of the IL coverage, the spectrum intensities decrease because of the formation of the IL adsorptive layer on the surface^31^.

### Quantum chemical studies

As known, the application of quantum chemical calculations is necessary to demonstrate the relation between the molecular/electronic structure of the inhibitor and its corrosion inhibiting efficiency^32^. Furthermore, the theoretical studies allow the optimum pre-selection of the inhibitor with the desired characteristics^33^.

The molecular geometry of the employed ionic liquid was optimized using the PM6 semiempirical method. The optimized structures were then re-optimized by ab initio Hartree–Fock method (HF) using 6–31 + G(d,p) basis set at Hartree–Fock level (HF)^34^, and by density functional theory (DFT) method using 6–31 + G(d,p) basis set. The values of the calculated bond length, bond angle, and dihedral angle using the DFT 6–31 + G (d,p) method are compiled in Table 5. All calculations were made by gaussian 09 W software using the B3LYP functional^35^. B3LYPutilizesBache’s three-parameter functional (B3) and comprises a combination of HF with DFT exchange terms and the Lee, Yang, and Parr (LYP) correlation functional^36^.

**Table 5:** Calculated bond length, bond angle, and dihedral angle using DFT 6–31 + G (d,p) method.

Tag	Symbol	NA	NB	NC	Bond	Angle	Dihedral
1	C						
2	C	1			1.3649388		
3	N	1	2		1.3826703	107.2577112	
4	C	3	1	2	1.3385765	108.2901377	0.1342003
5	H	1	2	3	1.0786527	130.5797538	− 179.7446671
6	H	2	1	4	1.0788841	130.7968854	179.5585425
7	H	3	1	2	1.0793932	162.0315182	− 179.4096889
8	C	4	3	1	1.4833922	125.7694245	− 178.2340041
9	H	8	4	3	1.0930267	106.6857276	− 17.4207763
10	H	8	4	3	1.0936370	106.8490727	− 132.2691668
11	C	8	4	3	1.5319684	112.6994544	104.9556159
12	H	11	8	4	1.0974252	109.5501526	58.6957637
13	H	11	8	4	1.0975405	109.2694754	− 58.0633895
14	C	11	8	4	1.5368842	111.5173836	− 179.7949393
15	H	14	11	8	1.0979581	109.3489727	− 57.6517339
16	H	14	11	8	1.0979929	109.3446071	58.5127304
17	C	14	11	8	1.5327518	112.4516784	− 179.5556948
18	H	17	14	11	1.0954488	111.3223019	60.4191595
19	H	17	14	11	1.0935363	110.7318492	− 179.8474457
20	H	17	14	11	1.0955026	111.3679463	− 60.0826591
21	N	3	1	2	1.3402623	72.5094756	0.0235677
22	C	21	3	1	1.4709147	125.9328112	− 179.6726306
23	H	22	21	3	1.0913211	109.4349618	− 119.9287493
24	H	22	21	3	1.0897125	108.9581759	− 0.2996535
25	H	22	21	3	1.0913515	109.4608757	119.3369532

**Figure 9:**
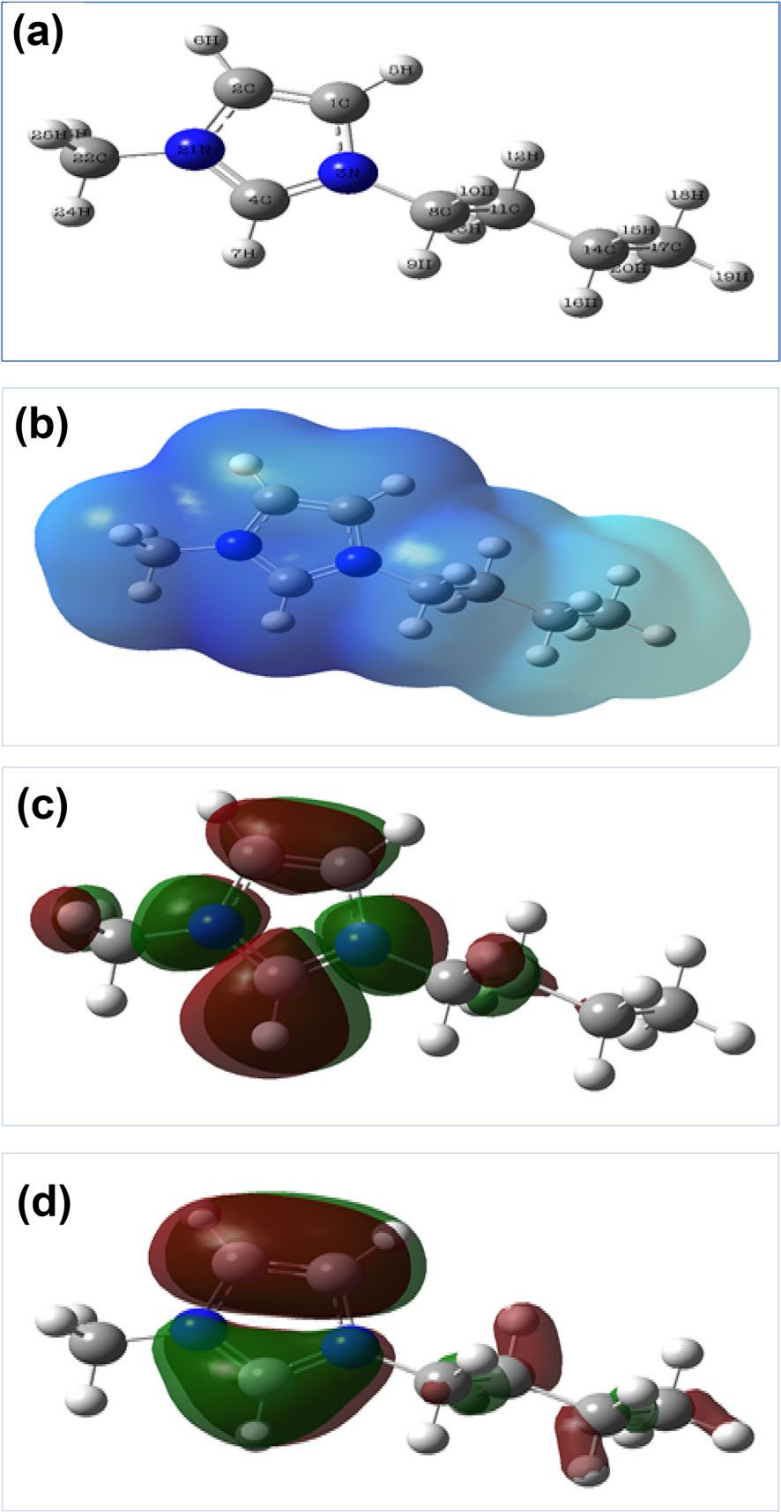
The frontier molecular orbital (**a**) optimized structure, (**b**) electrostatic potential (ESP), (**c**) HOMO, and (**d**) LUMO of the ionic liquid [BMIm]TfO.

The optimized molecular structure, the electrostatic potential feature, and the identical HOMO and LUMO electron density surfaces of the ionic liquid inhibitor, as demonstrated patterns by IL employing PM3 model chemistry, are symbolized in Fig. 9a–d. From the optimized structure, Fig. 9a it is seen the planar pentagon structure of the imidazolium ring. The electrostatic potential Fig. 9b it shows the electron cloud around the chemical structure. The sequential distribution of electrons of HOMO Fig. 9c shows information about the positions or locations that are significant in the system to accord the electrons to the contrasting orbital of the recipient molecule. On the other hand, the prevalence of electrons of LUMO Fig. 9d shows information about the molecule sites that are restricted to recognize the electron from a-convenient granter one^37^.

From Fig. 9c, it is obvious that the electron density of the HOMO is generally combined with the imidazolium cation in the ionic liquid structure indicating the contribution of the imidazolium ring in the adsorption of ionic liquid on the steel surface. This is consistent with the recently published results which indicated, based on quantum chemical studies, that the imidazolium ring is likely the crucial active site in imidazolium-based ionic liquids^38^. The calculated values of the quantum chemical parameters, E_HOMO_, E_LUMO,_ and ΔE, which are mainly affected by the electron interactions between the steel surface and the ionic liquid inhibitor were illustrated in Table 6. The E_HOMO_ value describes the ability of the inhibitor to donate electrons to the electrode surface, while the E_LUMO_ measures the capability to receive electrons into the LUMO molecule from a suited donor molecule^39–41^. The energy gap (ΔE), which is obtained from the difference between E_LUMO_ and E_HOMO_, is also a substantial factor that can be employed to detect the molecule's activity. Mainly, the minimal ΔE value is related to large inhibition efficiency and chemical reactivity^42–44^. This means the inhibitor on the surface more easily gives/receives electrons and subsequently the adsorption of the inhibitor on the surface becomes easier. The negative sign of E_HOMO_ refers to the physical adsorption nature of the ionic liquid inhibitor^45–47^.

**Table 6:** The calculated quantum chemical parameters in eV for the investigated inhibitor.

E_HOMO_	E_LUMO_	∆E
(eV)	(eV)	(eV)
**DFT/B3LYP/6-31G + (d,p)**		
− 11.740	− 5.067	6.673
**HF/6-31G + (d,p)**		
− 14.185	− 1.611	12.573
**Semi-empirical /PM6**		
− 14.506	− 4.509	9.996

In the light of the above results, it can be concluded that the employed ionic liquid is electrostatically adsorbed on the steel surface and the imidazolium cation is responsible for the observed inhibiting influence as the imidazolium ring is parallelly absorbed on the steel surface.

## Conclusions

[BMIm]TfO was examined as a possible corrosion inhibitor for carbon steel in chloride solutions. The results revealed the inhibiting action of [BMIm]TfO which increases as the concentration increases. The potentiodynamic polarization results showed that the ionic liquid is a mixed inhibitor as both cathodic and anodic processes were influenced. The inhibiting act of [BMIm]TfO is attributable to the adsorption of the imidazolium cations on the carbon steel surface establishing a barrier layer that protects the surface from the aggressive medium. The adsorption of the [BMIm]TfO was found to obey Langmuir isotherm. The free energy of adsorption revealed that the adsorption of the ionic liquid species occurs physically via the electrostatic interaction of the imidazolium cation with the charged centers on the electrode surface. The results proved that ionic liquids can be used as corrosion inhibitors for carbon steel in brine solutions with a high efficiency that enables them to compete with the commercial inhibitors currently used, despite their high price, they are considered competitive for more than one reason, including their effectiveness and the most important reason is that they are less toxic and therefore in line with the global trend to replace toxic materials. So, it could be currently used with potential effects and less toxic materials, which makes these materials ready to compete in the field of oil and gas industry.

## Data Availability

The datasets generated and/or analyzed during the current study are available in this published article.

## References

[1] Sun C, Liu J, Sun J, Lin X, Wang Y (2021). Probing the initial corrosion behavior of X65 steel in CCUS-EOR environments with impure supercritical CO_2_ fluids. Corros. Sci..

[2] Reda Y, Yehia HM, El-Shamy AM (2022). Microstructural and mechanical properties of Al-Zn alloy 7075 during RRA and triple aging. Egypt. J. Pet..

[3] Zohdy KM, El-Sherif RM, El-Shamy AM (2021). Corrosion and passivation behaviors of tin in aqueous solutions of different pH. J. Bio- Tribo-Corros..

[4] Sarkar TK, Saraswat V, Mitra RK, Obot IB, Yadav M (2021). Mitigation of corrosion in petroleum oil well/tubing steel using pyrimidines as efficient corrosion inhibitor: Experimental and theoretical investigation. Mater. Today Commun..

[5] Azam MA, Sukarti S, Zaimi M (2020). Corrosion behavior of API-5L-X42 petroleum/natural gas pipeline steel in South China Sea and Strait of Melaka seawaters. Eng. Fail. Anal..

[6] Abdel-Karim AM, El-Shamy AM, Reda Y (2022). Corrosion and stress corrosion resistance of Al Zn alloy 7075 by nano-polymeric coatings. J. Bio- Tribo Corros..

[7] Zhang S, Hou L, Du H, Wei H, Liu B, Wei Y (2020). A study on the interaction between chloride ions and CO_2_ towards carbon steel corrosion. Corros. Sci..

[8] Abbas MA, Bedair MA (2019). Adsorption and computational studies for evaluating the behavior of silicon based compounds as novel corrosion inhibitors of carbon steel surfaces in acidic media. Z. Phys. Chem..

[9] Tan B, Zhang S, Cao X, Anqing Fu, Guo L, Marzouki R, Li W (2022). Insight into the anti-corrosion performance of two food flavors as eco-friendly and ultra-high-performance inhibitors for copper in sulfuric acid medium. J. Colloid Interface Sci..

[10] Tan B, Lan W, Zhang S, Deng H, Qiang Y, Fu A, Ran Y, Xiong J, Marzouki R, Li W (2022). *Passiflora edulia* Sims leaves extract as renewable and degradable inhibitor for copper in sulfuric acid solution. Colloids Surf. A.

[11] El-Shamy AM, El-Hadek MA, Nassef AE, El-Bindary RA (2020). Optimization of the influencing variables on the corrosion property of steel alloy 4130 in 3.5 wt.% NaCl solution. J. Chem..

[12] El-Shamy AM, El-Hadek MA, Nassef AE, El-Bindary RA (2020). Box-Behnken design to enhance the corrosion resistance of high strength steel alloy in 3.5 wt.% NaCl solution. Mor. J. Chem..

[13] Attia H, Mohamed Nawal H, Soad M, Samar M (2007). Phytochemical and pharmacological studies on *Convolvulus fatmensi*s Ktze. J. Nat. Remed..

[14] Bhaskaran Y, Pancharatana PD, Sharma RK, Kaur G, Lata S, Singh G (2021). To evaluate an ionic liquid for anticorrosive impact on iron–carbon steel: Synthesis, computational and experimental mechanism. Chem. Pap..

[15] Mohsenifar F, Jafari H, Sayin K (2016). Investigation of thermodynamic parameters for steel corrosion in acidic solution in the presence of N, N′-Bis (phloroacetophenone)-1, 2 propanediamine. J. Bio- Tribo- Corros..

[16] Reda Y, El-Shamy AM, Eessaa AK (2018). Effect of hydrogen embrittlement on the microstructures of electroplated steel alloy 4130. Ain Shams Eng. J..

[17] Gad EA, El-Shamy AM (2022). Mechanism of corrosion and microbial corrosion of 1,3-dibutyl thiourea using the quantum chemical calculations. J. Bio- Tribo- Corros..

[18] Xu X, Singh A, Sun Z, Ansari KR, Lin Y (2017). Theoretical, thermodynamic and electrochemical analysis of biotin drug as an impending corrosion inhibitor for mild steel in 15% hydrochloric acid. R. Soc. Open Sci..

[19] Hossain N, Asaduzzaman Chowdhury M, Kchaou M (2021). An overview of green corrosion inhibitors for sustainable and environment friendly industrial development. J. Adhes. Sci. Technol..

[20] Ardakani EK, Kowsari E, Ehsani A, Ramakrishna S (2021). Performance of all Ionic Liquids as the eco-friendly and sustainable compounds in inhibiting corrosion in various media: A comprehensive review. Microchem. J..

[21] El-Shamy AM, Zakaria K, Abbas MA, El Abedin SZ (2015). Anti-bacterial and anti-corrosion effects of the ionic liquid 1-butyl-1-methylpyrrolidinium trifluoromethylsulfonate. J. Mol. Liq..

[22] Mouneir SM, El-Hagrassi AM, El-Shamy AM (2022). A review on the chemical compositions of natural products and their role in setting current trends and future goals. Egypt. J. Chem..

[23] Sherif ESM, Abdo HS, El Abedin SZ (2015). Corrosion inhibition of cast iron in Arabian gulf seawater by two different ionic liquids. Materials.

[24] Shehata MF, El-Shamy AM, Zohdy KM, Sherif ESM, Zein El Abedin S (2020). Studies on the antibacterial influence of two ionic liquids and their corrosion inhibition performance. Appl. Sci..

[25] Musa AY, Mohamad AB, Kadhum AAH, Takriff MS, Ahmoda W (2012). Quantum chemical studies on corrosion inhibition for series of thio compounds on mild steel in hydrochloric acid. J. Ind. Eng. Chem..

[26] Abdel-Karim AM, El-Shamy AM (2022). A review on green corrosion inhibitors for protection of archeological metal artifacts. J. Bio- Tribo- Corros..

[27] Kabel KI, Zakaria K, Abbas MA, Khamis EA (2015). Assessment of corrosion inhibitive behavior of 2-aminothiophenol derivatives on carbon steel in 1 M HCl. J. Ind. Eng. Chem..

[28] Joshi SJ, Deshmukh A, Sarma H (2021). Biotechnology for Sustainable Environment.

[29] Verma C, Singh A, Pallikonda G, Chakravarty M, Quraishi MA, Bahadur I, Ebenso EE (2015). Aryl sulfonamidomethylphosphonates as new class of green corrosion inhibitors for mild steel in 1 M HCl: Electrochemical, surface and quantum chemical investigation. J. Mol. Liq..

[30] Bakr AA, Zakaria K, Abbas MA, Hamdy A (2016). Amphistegina media filtration as pretreatment of SWRO desalination unit for producing different salinities to study the corrosion behavior of various materials. Desalin. Water Treat..

[31] Ouakki M, Galai M, Rbaa M, Abousalem AS, Lakhrissi B, Touhami ME, Cherkaoui M (2020). Electrochemical, thermodynamic and theoretical studies of some imidazole derivatives compounds as acid corrosion inhibitors for mild steel. J. Mol. Liq..

[32] Soliman SA, Metwally MS, Selim SR, Bedair MA, Abbas MA (2014). Corrosion inhibition and adsorption behavior of new Schiff base surfactant on steel in acidic environment: Experimental and theoretical studies. J. Ind. Eng. Chem..

[33] Farag AA, Ismail AS, Migahed MA (2018). Environmental-friendly shrimp waste protein corrosion inhibitor for carbon steel in 1 M HCl solution. Egypt. J. Pet..

[34] Mashuga ME, Olasunkanmi LO, Adekunle AS, Yesudass S, Kabanda MM, Ebenso EE (2015). Adsorption, thermodynamic and quantum chemical studies of 1-hexyl-3-methylimidazolium based ionic liquids as corrosion inhibitors for mild steel in HCl. Materials.

[35] Abbass M, Mohammed KZ, Hamdy A (2012). Adsorption properties and inhibitive effect of antibacterial drug on carbon steel corrosion in HCl medium. Biosci. Biotechnol. Res. Asia..

[36] Hameed RSA, Ismail EA, Al-Shafey HI, Abbas MA (2020). Expired indomethacin therapeutics as corrosion inhibitors for carbon steel in 1.0 M hydrochloric acid media. J. Bio- Tribo-Corros..

[37] Gece G (2008). The use of quantum chemical methods in corrosion inhibitor studies. Corros. Sci..

[38] Frisch, M. *et al*. Gaussian 09, Revision A. 01 (2009).

[39] Lee C, Yang W, Parr RG (1988). Development of the Colle-Salvetti correlation-energy formula into a functional of the electron density. Phys. Rev. B..

[40] Tian G, Yuan K (2021). Adsorption and inhibition behavior of imidazolium tetrafluoroborate derivatives as green corrosion inhibitors for carbon steel. J. Mol. Model..

[41] Singh AK, Chugh B, Saha SK, Banerjee P, Ebenso EE, Thakur S, Pani B (2019). Evaluation of anti-corrosion performance of an expired semi-synthetic antibiotic cefdinir for mild steel in 1 M HCl medium: An experimental and theoretical study. Results Phys..

[42] Zohdy KM, El-Sherif RM, Ramkumar S, El-Shamy AM (2021). Quantum and electrochemical studies of the hydrogen evolution findings in corrosion reactions of mild steel in acidic medium. Upstream Oil Gas Technol..

[43] Karthikaiselvi R, Subhashini S (2014). Study of adsorption properties and inhibition of mild steel corrosion in hydrochloric acid media by water-soluble composite poly (vinyl alcohol-o-methoxy aniline). J. Assoc. Arab Univ. Basic Appl. Sci..

[44] Tang Y, Yang X, Yang W, Wan R, Chen Y, Yin X (2010). A preliminary investigation of corrosion inhibition of mild steel in 0.5 M H_2_SO_4_ by 2-amino-5-(n-pyridyl)-1, 3, 4-thiadiazole: Polarization, EIS and molecular dynamics simulations. Corros. Sci..

[45] Farag HK, El-Shamy AM, Sherif EM, El Abedin SZ (2016). Sonochemical synthesis of nanostructured ZnO/Ag composites in an ionic liquid. Z. Phys. Chem..

[46] Abbas MA, Zakaria K, El-Shamy AM, El Abedin SZ (2019). Utilization of 1-butylpyrrolidinium chloride ionic liquid as an eco-friendly corrosion inhibitor and biocide for oilfield equipment: Combined weight loss, electrochemical and SEM studies Z. Phys. Chem..

[47] El-Shamy AM, Abdel Bar MM (2021). Ionic liquid as water soluble and potential inhibitor for corrosion and microbial corrosion for iron artifacts. Egypt. J. Chem..

