# Author Correction: Adsorption, thermodynamic, and quantum chemical investigations of an ionic liquid that inhibits corrosion of carbon steel in chloride solutions

**DOI:** 10.1038/s41598-022-22010-9

**Published:** 2022-10-11

**Authors:** Mohamed A. Abbas, Amr S. Ismail, K. Zakaria, A. M. El-Shamy, S. Zein El Abedin

**Affiliations:** 1 Petroleum Applications Department, Egyptian Petroleum Research Institute, P.B. 11727, Nasr City, Cairo Egypt; 2 Petrochemicals Department, Egyptian Petroleum Research Institute, P.B. 11727, Nasr City, Cairo Egypt; 3 Analysis and Evaluation Department, Egyptian Petroleum Research Institute, P.B. 11727, Nasr City, Cairo Egypt; 4 Electrochemistry and Corrosion Laboratory, Physical Chemistry Department, National Research Centre, Dokki, 12622 Cairo Egypt

Correction to: *Scientific Reports* 10.1038/s41598-022-16755-6, published online 22 July 2022

In the original version of this Article, A. M. El Shamy and S. Zein El Abedin were incorrectly affiliated with ‘Petrochemicals Department, Egyptian Petroleum Research Institute, P.B. 11,727, Nasr City, Cairo, Egypt.’ Their correct affiliation is listed below.

Electrochemistry and Corrosion Laboratory, Physical Chemistry Department, National Research Centre, Dokki, 12622, Cairo, Egypt.

The original Article has been corrected.

