# Author Correction: Adsorption, thermodynamic, and quantum chemical investigations of an ionic liquid that inhibits corrosion of carbon steel in chloride solutions

**DOI:** 10.1038/s41598-023-28979-1

**Published:** 2023-02-06

**Authors:** Mohamed A. Abbas, Amr S. Ismail, K. Zakaria, A. M. El Shamy, S. Zein El Abedin

**Affiliations:** 1 Petroleum Applications Department, Egyptian Petroleum Research Institute, Nasr City, P.B. 11727, Cairo, Egypt; 2 Petrochemicals Department, Egyptian Petroleum Research Institute, Nasr City, P.B. 11727, Cairo, Egypt; 3 Analysis and Evaluation Department, Egyptian Petroleum Research Institute, Nasr City, P.B. 11727, Cairo, Egypt; 4 Electrochemistry and Corrosion Laboratory, Physical Chemistry Department, National Research Centre, Dokki, Cairo, 12622 Egypt

Correction to: *Scientific Reports* https://doi.org/10.1038/s41598-022-16755-6, published online 22 July 2022

The original version of this Article contained errors in the affiliations.

Mohamed A. Abbas was incorrectly affiliated with ‘Petrochemicals Department, Egyptian Petroleum Research Institute, P.B. 11727, Nasr City, Cairo, Egypt.’

In addition, Amr S. Ismail was incorrectly affiliated with ‘Petroleum Applications Department, Egyptian Petroleum Research Institute, P.B. 11727, Nasr City, Cairo, Egypt.’

Finally, K. Zakaria was incorrectly affiliated with ‘Petrochemicals Department, Egyptian Petroleum Research Institute, P.B. 11727, Nasr City, Cairo, Egypt.’

The correct affiliations are listed below:

**Mohamed A. Abbas:** Petroleum Applications Department, Egyptian Petroleum Research Institute, P.B. 11727, Nasr City, Cairo, Egypt.

**Amr S. Ismail:** Petrochemicals Department, Egyptian Petroleum Research Institute, P.B. 11727, Nasr City, Cairo, Egypt.

**K. Zakaria:** Analysis and Evaluation Department, Egyptian Petroleum Research Institute, P.B. 11727, Nasr City, Cairo, Egypt.

The original Article has been corrected.

